# Janani Surakhya Yojana and ‘At Birth’ Immunization: A Study in a Tertiary Level Health Center

**DOI:** 10.4103/0970-0218.58398

**Published:** 2009-10

**Authors:** DM Satapathy, D Shobha Malini, TR Behera, SSS Reddy, RM Tripathy

**Affiliations:** Department of Community Medicine, MKCG Medical College, Berhampur, India

## Introduction

The ‘Janani Surakshya Yojana’ (JSY) is an ambitious step under NRHM which is introduced on 12^th^ Apr 2005 to reduce Neonatal and Maternal mortality by promoting institutional deliveries as well as better ante natal care and post natal care through Accredited Social Health Activist (ASHA).(1)

Institutional deliveries will not only facilitate safe delivery but will also identify neonates who need special care. The safe delivery process conducted in an institution will have a definite impact of reduction of maternal mortality. Delivery of a newborn in an Institution also provides an opportunity to the health care system to administer at birth immunization. Immunization of a newborn with BCG vaccine enhances the efficacy of the vaccine by avoiding the interference with atypical mycobacterium which can infect the child during the post neonatal period.(2)

Likewise, administration of “0 dose” of OPV leads to early colonization of the intestinal tract with the attenuated vaccine virus which can act as a barrier to the wild polio virus.(3) At birth immunization is a important preventive measure however the impact of JSY scheme on at birth immunization practice especially in tertiary level health center has not yet been documented.

The present study was undertaken with the following objectives: a) To study the pattern of ‘at birth’ immunization after the implementation of JSY. b) To compare it with the status of previous year before implementation of JSY. c) To study the perception of parents regarding ‘at birth’ immunization.

## Materials and Methods

The study was cross-sectional one and the data was collected by a pre-designed schedule from the parents of all the babies attending the immunization clinic for BCG and ‘0’dose OPV immunization from 1^st^ April 2007 to 30^th^ September 2007 in the Epidemiology out-patient department (OPD) of Department of Community Medicine, MKCG Medical College Hospital, Brahmapur, Orissa. The JSY scheme was implemented in this tertiary level health care center from 12^th^ May 2006, but the operationalization of the scheme came into effect from the month of Dec 2006. As the financial year of the government starts from 1^st^ Apr of every year, the comparison of ‘at birth’ immunization were from 1^st^ Apr 2006 to 30^th^ Sep 2006 as pre JSY scheme and between the same period of the year 2007 i.e. 1^st^ Apr to 30^th^ Sep 2007 as the period of JSY scheme operationalization. The data on those periods were collected by record-survey from the register of the immunization clinic. The parents were also interviewed for perception regarding at birth immunization and the data were analyzed in the Department of Community Medicine, MKCG Medical College Hospital.

## Results

**Figure 1:**
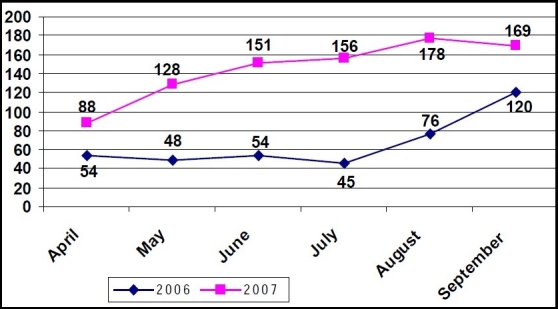
Comparison of month-wise distribution of ‘at birth’ immunization in year 2006 and 2007. Median average monthly ‘at birth’ immunization in 2006 = 54. Median average monthly ‘at birth’ immunization in 2007 = 153

The study revealed that during 1^st^ April to 30^th^ September 2006, the total no of ‘at birth’ immunization carried out was 397 (out of the 1699 babies attended for immunization) with a median average monthly administration of 54. After the implementation of JSY Scheme in this Medical College Hospital, the total number of ‘at birth’ immunization carried out in the same months of 2007 was 870 (out of the 2332 babies attended for immunization) with a median average monthly administration of 153 [Figure 1].

In 2007 out of the 870 immunized babies 468 (53.8%) were male and 402 (46.2%) were female and regarding their residential status 32% were from urban and 68% were from rural area, where as in 2006 the male babies attended for immunization were 237 (59.7%) and female babies were 160 (40.3%) and their residential status are 45.8% from urban and 54.2% from rural area. The total number of deliveries in the Hospital during April-Sept 2006 was 714 and at birth immunization given was 397 i.e. 55.6% of the newborns turned out for at birth immunization, whereas in 2007 during the same period 870 new borns out of 920 were immunized at birth i.e. about 94.5%, which showed a drastic increase of at birth immunization.

In 2007, a total of 769 (88.4%) babies attended the immunization clinic within 7 days of birth where as in 2006 during the same period, a total of 206 (51.9%) babies attended within 7 days of birth for ‘at birth’ immunization. The difference in number of at birth immunization in pre and post JSY period was found to be highly significant (*P* < 0.001) [Table 1].

**Table 1:** Impact of JSY over timing of at birth immunization

Year	At birth immunization within 7 days	At birth immunization after 7 days	Total
2006	206	191	397
2007	769	101	870
Total	975	292	1267

χ^2^= 201 *P*< 0.001

Regarding perception of parents on ‘at birth’ immunization, 49.4% parents from urban and 56.2% parents from rural area knew about it from ASHA, 14.3% parents from urban and 28.4% parents from rural area were knew it from Health Worker Female/Anganwadi Worker. 25.1% parents from urban and only 8.8% parents from rural area were self-aware regarding at birth immunization [Table 2].

**Table 2:** Perception of parents on at birth immunization

Perception of parents on at birth immunization number (%)	
Motivator for at birth immunization (Urban area)	
Accredited social health activist	138 (49.4)
Auxiliary nurse midwife / Anganwadi worker	40 (14.3)
Family member/self aware	70 (25.1)
Others	31 (11.1)
Motivator for at birth immunization (Rural area)	
ASHA	332 (56.2)
Auxiliary nurse midwife / Anganwadi worker	168 (28.4)
Family member/self aware	52 (8.8)
Others	39 (6.6)
Vaccines given at birth	
BCG	642 (73.8)
OPV	791 (90.9)
Hep-B	72 (8.3)
Don't know	79 (9.0)
At birth immunization prevents	
Tuberculosis	230 (26.4)
Polio	654 (75.1)
Hepatitis-B	72 (8.3)
Don't know	216 (24.8)

642 (73.8%) parents knew that BCG is given at birth and 791 (90.91%) knew that OPV is given at birth, but only 230 (26.43%) and 654 (75.17%) parents knew that these vaccines prevent tuberculosis and polio, respectively [Table 2].

## Discussion

The ‘at birth’ immunization from April to September from 2006 to 2007 increased significantly (*P* < 0.001) after implementation of JSY. It may be due to rise in institutional deliveries which are motivated by the monetary incentive given to the mother. At birth immunization during early neonatal period is increased significantly from 51.9 to 88.4% as mostly ASHA's accompany the mother and ASHA gets her incentive after immunizing the child. Similar studies done in Afghanistan by A. Hadi *et al*. reveals that institutional deliveries increases where there is intensive community mobilization, provision of free services, transport facilities at night, incentive to health providers and quality of services which are the key factors for rise in institutional delivery.(4)

The total number of deliveries in the hospital during April-Sept 2006 to 2007 increased from 714 to 920 and the increase in at birth immunization was seen from 55.6 to 94.5%, which showed a dramatic increase. A similar study was conducted at Bongaigaon district and the institutional deliveries conducted during the period of April to August 2006 were 601, whereas during the same period in 2007 it was 1524.(5)

Most cases are from rural areas as it is a tertiary level health care; the patients from nearby areas are referred to this hospital. ASHA's do work of community mobilization and motivation. Similar studies by Huang J and Tembo KC have found that provision of services alone can not raise the need and utilization of services unless there is motivation.(6,7)

After the implementation of JSY scheme, ASHA's have been successful in promoting institutional deliveries. Family members and mothers are convinced to go for institutional deliveries, so effective promotion among pregnant women and their family members played a role for rise in institutional deliveries.(8)

One-fourth of the parents from urban area were self-aware regarding at birth immunization, which was very low among rural parents. This may be due to literacy level. In states like Tamil Nadu and Kerala awareness about maternal health issue is high and citizens demand more. Literacy also plays a key role in rendering maternal health services.(9)

Though the number is less, it is very encouraging that some parents knew about Hepatitis-B vaccination during ‘at birth’ immunization.

## Conclusion

JSY has significant impact on institutional delivery and Accredited social health activist/Anganwadi worker are the main motivator for ‘at birth’ immunization.

Monetary benefit of ASHA is the main factor which brings people for at birth immunization, not the vaccines which prevent the disease. Therefore, steps to create social mobilization among the community to avail not only the benefits but also on the objective of JSY should be immediately taken up.

The thrust of the NRHM was to establish a fully functional, community owned, decentralized health delivery system. The implementation of JSY scheme will surely have an impact on indicators like MMR, IMR due to the rise of institutional delivery.

The innovative engagement of human resources as per need and the arrangements for incentives at each level will help to build up a role model of public health delivery system.

## References

[1] Kishore J (2007). J Kishore's Textbook of National Health Program.

[2] Vijayalakshmi V, Sadhana PC, Murthy KJR (1993). Non-tuberculous mycobacterial infections. Indian J Tuberc.

[3] Khare S, Kumari S, Singh Nagpal I, Sharma D, Verghese T (1993). Oral Polio Vaccination in Infants: Beneficial Effect of Additional Dose at Birth. Indian J Pediatr.

[4] Hadi A, Rahman T, Khuram D, Ahmed J, Alam A Raising institutional delivery in war-torn communities: Experience of BRAC in Afghanistan. Asia Pacific Journal of Family Medicine.

[5] A Brief Progress Report of Different Schemes Under NRHM, Bongaigaon district.

[6] Huang J, Xue Y, Jia Y, Xue J (1994). Evaluation of a health education programme in china to increase breast-feeding rates. Health Promot Int.

[7] Tembo KC (1995). Grass- root health education strategies in Malwai. J R Soc Health.

[8] Kobinsky MA, Campbell O, Heichelheim J (1999). Organizing delivery care: What works for safe motherhood?. Bull World Health Organ.

[9] Indian Together: Who cries when mother dies-21^st^ Nov.

